# IKKε Knockout Prevents High Fat Diet Induced Arterial Atherosclerosis and NF-κB Signaling in Mice

**DOI:** 10.1371/journal.pone.0064930

**Published:** 2013-05-31

**Authors:** Changchun Cao, Yifan Zhu, Wen Chen, Liangpeng Li, Yongchao Qi, Xiaodi Wang, Ye Zhao, Xin Wan, Xin Chen

**Affiliations:** 1 Division of Thoracic and Cardiovascular Surgery, Department of surgery, Nanjing First Hospital, Nanjing Medical University, Nanjing, Jiangsu, China; 2 Division of Nephrology, Department of medicine, Nanjing First Hospital, Nanjing Medical University, Nanjing, Jiangsu, China; Charité, Campus Benjamin Franklin, Germany

## Abstract

**Aims:**

Atherosclerosis is a public health concern affecting many worldwide, but its pathogenesis remains unclear. In this study we investigated the role of IKKε during the formation of atherosclerosis and its molecular mechanism in the mouse aortic vessel wall.

**Methods and Results:**

C57BL/6 wild-type or IKKε knockout mice bred into the ApoE knockout genetic background were divided into 4 groups: (1) wild-type (WT), (2) ApoE knockout (AK), (3) IKKε knockout (IK), (4) or both ApoE and IKKε knockout (DK). Each group of mice were fed with a high fat diet (HFD) for 12 weeks from 8 weeks of age. Immunohistochemistry and Western blotting analysis demonstrated obvious increases in the expression of IKKε in the AK group compared with the WT group, especially in the intima. Serum lipid levels were significantly higher in the AK and DK groups than in the other two groups. Staining with hematoxylin-eosin and Oil Red, as well as scanning electron microscopy revealed less severe atherosclerotic lesions in the DK group than in the AK group. Immunofluorescence and Western blot analysis demonstrated obvious increases in the expression of NF-κB pathway components and downstream factors in the AK group, especially in the intima, while these increases were blocked in the DK group.

**Conclusion:**

The knockout of IKKε prevented significant atherosclerosis lesions in the mouse aorta from in both wild-type and ApoE knockout mice fed a HFD, suggesting that IKKε may play a vital role in HFD-induced atherosclerosis and would be an important target for the treatment of atherosclerosis.

## Introduction

Atherosclerosis, a progressive pathological disorder underlying cardiovascular diseases, is the major cause of mortality and morbidity in industrialized societies and is characterized by a large number of risk factors with multiple pathogenesis hypotheses [1]–[2]. The initiation and progression of atherosclerosis have been attributed to a chronic inflammatory process due to high fat diet (HFD)-induced lipid accumulation in the subendothelial space and lipid peroxidation-promoted endothelial cell activation. Activated endotheial cells modulate the expression of many different cell adhesion molecules, chemotactic factors and proinflammatory cytokines, which contribute to the recruitment and migration of both monocytes and smooth muscle cells in the vessel wall [3]–[7]. However, the exact underlying mechanisms are still far from being fully understood.

The NF-κB transcription factor has been implicated in the pathogenesis of atherosclerosis as an important regulatory factor of inflammation [8]. The activation of NF-κB is modulated by the IκB kinase (IKK) complex. NF-κB binds to its inhibitory unit (IκB) in the cytoplasm of resting cells as an inactive complex. When triggered by various stimulatory signals, such as cytokines, oxidants, mitogens, bacterial and viral products, the phosphorylation and degradation of IκBs occur via combination with the IKK complex, which can then activate NF-κB. The liberated NF-κB then translocates into the nucleus where it activates the expression of downstream target genes, including those which encode proinflammatory cytokines, cell adhesion molecules and chemotactic factors, and contributes to the acceleration of atherosclerosis [9]–[13]. Past studies have shown that IKKβ is the main component of the IKK complex which serves as a kinase for the phosphorylation and ubiquitination of IκBs [14]. Although numerous studies have concentrated on members of the IKK complex, a conclusion has still not been reached that the inhibition of IKKβ cannot prevent the development of atherosclerosis [15]–[16].

A new member of the IKK complex, IKKε (IKK-i), with structural similarity to IKKβ was identified several years ago [17]–[20]. In 2009, a report indicated that a HFD induces the expression of IKKε and increases the activation of NF-κB in the mouse liver and adipose tissue, while knockout of the IKKε gene protects against HFD-induced obesity and chronic inflammation of both liver and adipose tissue. This observation provided us with a new link between IKKε and HFD-induced atherosclerosis [21]. Furthermore, several studies have also suggested the potential role of IKKε in inflammatory pain, rheumatoid arthritis and osteoarthritis through the NF-κB activation cascade [22]–[23]. In this study, we therefore investigated whether IKKε plays a critical role in the initiation and progression of atherosclerosis through activation of the NF-κB pathway.

## Methods

### 1. Mice

IKKε knockout mice (B6.Cg-Ikbke^tm1Tman^/J), purchased from the Jackson laboratory (Bar Harbor, ME, USA), underwent rederivation to achieve pathogen-free status in the Model Animal Research Center of Nanjing University (Nanjing, China). Male wild-type (WT) control (C57BL/6) mice and ApoE knockout mice were obtained from the Model Animal Research Center of Nanjing University (Nanjing, China) at the age of 8 weeks. IKKε knockout mice were bred into the ApoE knockout genetic background to gain the DK group of mice. Each group of C57BL/6 mice were fed a HFD consisting of 60% of calories from fat (5.5% soybean oil, 54.5% lard, Research Diets 12492) for 12 weeks from 8 weeks of age. All mice were housed in specific pathogen-free box cages at 23±2°C and 60±10% humidity, with a 12-hour light/12-hour dark cycle and free access to food and water. The mice were sacrificed at week 12 of the experimental period. All animal procedures were performed in compliance with the Institute of Laboratory Animal Research Guide for the Care and Use of Laboratory Animals of the National Institutes of Health and approved by the Institutional Animal Care and Use Committee of Nanjing Medical University.

### 2. Lipid Analysis

After being fed the HFD for 12 weeks, mice were fasted for 12 h before anesthetization by 2.5% isoflurane inhalation. Each mouse was maintained on 1% isoflurane in an oxygen/air mixture using a gas anesthesia mask in a stereotaxic frame, and the toe pinch reflex, muscular relaxation, and respiration rates were monitored to determine that adequate anesthesia was administered. In order to minimize pain, mice also were given an intraperitoneal injection of pentobarbital (50 mg/kg body weight), and the adequacy of anesthesia was evaluated by monitoring hind limb reflexes. Blood samples were obtained from the retro-orbital plexus. Total cholesterol (TC) and triglycerides (TG) in the serum were determined using colorimetric enzymatic assays that were adapted to the 96-well format (Infinity Total Cholesterol Reagent or Infinity Triglyceride Reagent, Sigma).

### 3. Tissue Collection and Morphological Analysis

After euthanasia, mouse heart tissues of the atrioventricular valve region with attached aortic roots and ascending aorta were collected, fixed in 4% formalin for 48 h and then embedded in paraffin. Serial aortic sections (3 µm thickness) were then prepared and stained with hematoxylin and eosin (H&E). To assess the atherosclerotic lesions, the aortas were removed and frozen in Optimum Cutting Temperature (OCT) compound. Consecutive frozen aortic sections (10 µm thickness) were prepared and stained with Oil Red O for 10 min and counterstained with hematoxylin at room temperature. The total length of the aortic vessel wall from the aortic sinus to the abdominal aorta was also taken out entirely, opened longitudinally with the intima towards the outside and stained with Oil Red O for 10 min. Adobe Photoshop was used to quantify the plaque areas of the digitized microscopic images through the measuring of the Oil Red O stained area. For scanning electron microscopy, mouse ascending aorta samples were fixed in 2.5% glutaraldehyde solution for more than 24 h and then washed in phosphate buffered saline (pH = 7.4), post-fixed in 1% osmium tetroxide and dehydrated in increasing concentrations of acetone. Ultrathin sections of ∼60 nm thickness were collected in copper grids and stained with uranylacetate and lead citrate. Ten samples were chosen randomly from each group for examination under a Model S-3000N scanning electron microscope (Hitachi High-Technologies, Tokyo, Japan).

### 4. Immunohistochemical Staining

Tissues collected for morphological analysis with aortic roots and ascending aorta were prepared as 3 µm thick serial paraffin embedded sections and rehydrated in graded alcohol. The sections were treated with 3% hydrogen peroxide for 15 min to block endogenous peroxidase activity and incubated in buffered normal horse serum to prevent non-specific binding of antibodies. The sections were then incubated separately for 14 h with antibodies against IKKε (1∶1000; Novus Biologicals, Littleton, USA), followed by incubation with horseradish peroxidase (HRP)-conjugated goat anti-rabbit IgG (Beijing Zhongshan Biotechnology Co., Beijing, China) for 1 h at 37°C in a humidified box. The signal of each antibody was developed using the substrate diaminobenzidine (DAB, Beijing Zhongshan Biotechnology Co.). Sections were counterstained with hematoxylin, and photomicrographs were taken using an Olympus BX-URA2 camera.

### 5. Western Blotting Analysis

Total protein samples (50 µg) were extracted from the total length of the aorta from mice of each group and separated by SDS-PAGE. The proteins were transferred to polyvinylidene fluoride (PVDF) membranes (Millipore, Billerica, MA, USA), which were washed twice in Tris-buffered saline (TBS) with Tween® diluted 1∶1000 (TBST; Promega, Madison, WI, USA), for 10 min each time, and blocked with TBS containing 5% non-fat milk powder for 1 h. The membranes were probed with the following primary antibodies in TBS with Tween plus 5% milk overnight at 4°C: anti-IKKε (1∶200; Novus); anti-phosphorylated p50 (1∶200; KeyGEN, Nanjing, China); anti-phosphorylated p65 (1∶200; Cell Signaling, Beverly, MA, USA); anti-p65 (1∶200), anti-p50 (1∶200), anti-IκB-α (1∶200), anti-IL-18 (1∶200), anti-VEGF (1∶200) and anti-GAPDH (1∶5000) from Santa Cruz Biotechnology. The following day, PVDF membranes were washed with TBST four times, for 10 min each time. Subsequently, the PVDF membranes were incubated with appropriately diluted peroxidase-conjugated goat anti-rabbit secondary antibodies (1∶1000; Beijing ZhongShan Biotechnology Co.) at room temperature for 1 h. Specific proteins were detected using an ECL reagent (GE Healthcare, Piscataway, NJ, USA) and captured on Hyperfilm (Amersham, GE Healthcare). The results were then analyzed through the Image J software for semiquantitation of the mean gray value of each blot. Thereafter, the SPSS statistical software was used to perform one-way analysis of variance (ANOVA) to detect the differences among groups of mice. All presented results are representative of at least three independent experiments.

### 6. Immunofluorescence Staining

Tissues collected for morphological analysis with aortic roots and ascending aorta were prepared as 4 µm thick serial OCT embedded cryosections. The sections were fixed in 4% paraformaldehyde for 30 min, washed in PBS, and then incubated in buffered normal goat serum to prevent non-specific binding of antibodies for 1 h at room temperature. The sections were then incubated separately overnight with antibodies against P50, P65 and VEGF (1∶100; Santa Cruz Biotechnology, CA, USA), followed by incubation with Alexa Fluor 592 goat anti-rabbit IgG (1∶200; Invitrigen, Carlsbad, CA, USA) for 1 h at 37°C in a humidified box. Thereafter, the sections were washed in PBS and counterstained with Hoechst DNA dye (10 µg/ml;Sigma, St. Louis, USA) to illuminate nuclei. Photomicrographs were taken at random using an Olympus BX-URA2 camera in 4 sections per mouse sample.

### 7. Statistical Analysis

Data are presented as means ± SD. Differences among groups were analyzed by one-way ANOVA using SPSS13.0 (SPSS Inc, Chicago, IL, USA) software. Statistical differences between groups were analyzed by the least siginificant difference method. The significance level was set at *P*<0.05.

## Results

### 1. Activation of IKKε Contributes to HFD-induced Atherosclerosis

**Figure 1:**
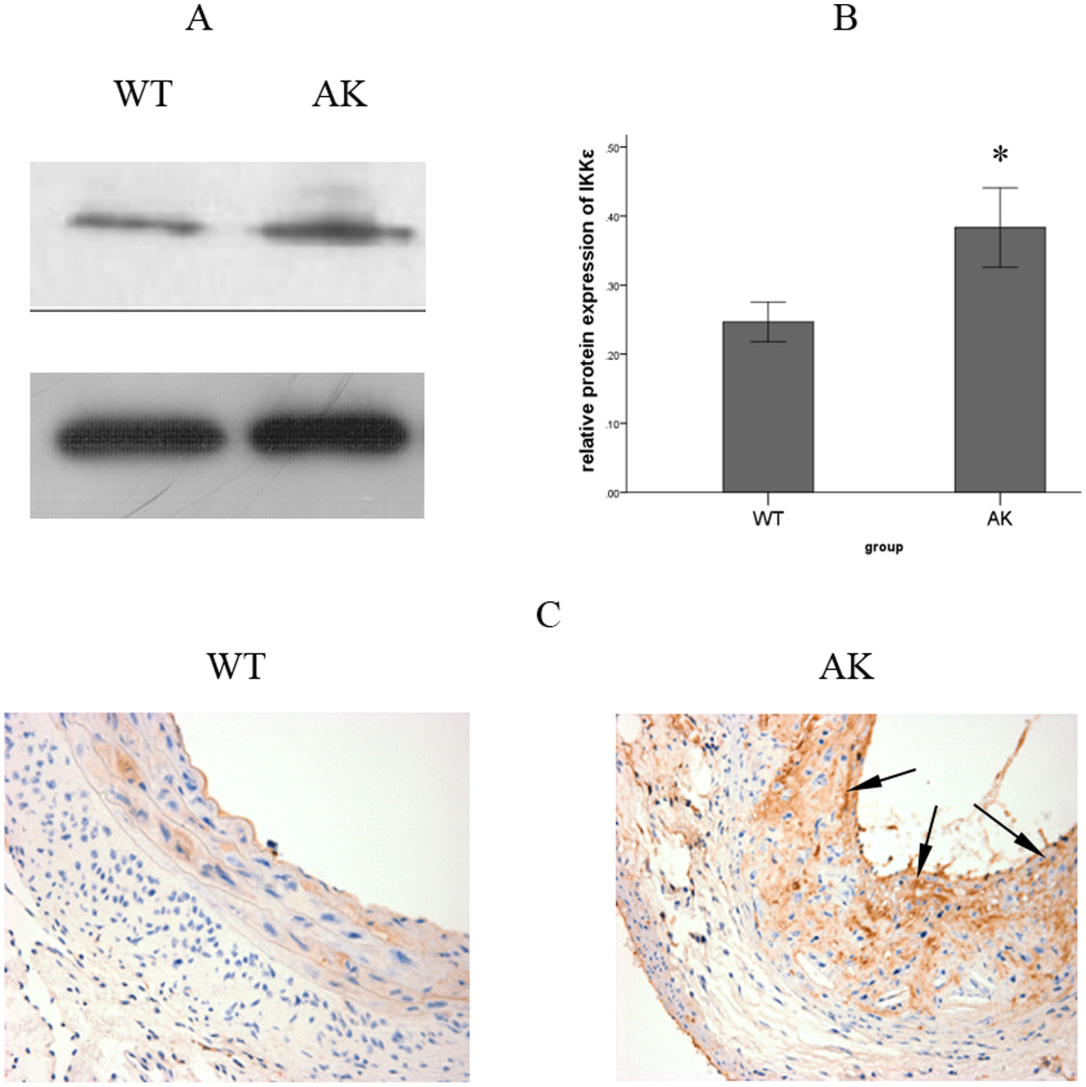
Protein expression and localization of IKKε after a HFD feeding. (*A*) IKKε was detected by Western blotting. (*B*) IKKε protein expression was normalized to GAPDH in the aortic vessel wall. Expression of IKKε was notably higher in the AK group than in the WT group. Values are means ± SD; n = 9 per group. Densitometric data are from one representative experiment of three separate experiments. *P<0.05. (*C*) Immunohistochemistry staining of IKKε also showed that the HFD-induced expression of IKKε was mostly distributed in the intima area of the aortic vessel wall (arrows, 400×).

A surprising increase in the expression of IKKε was first discovered in the AK group compared with the WT group through the application of Western blot analysis (*Figure 1A-B*). Similar results were obtained by immunohistochemical staining for IKKε. In addition to the increase in the expression of IKKε in the AK group, the expression of IKKε was also noted to be distributed mostly in the intimal area of the aortic vessel wall (*Figure 1C*). No staining was observed in the DK or IK group at the same time (*Figure S1*), confirming the specificity of the IKKε antibody.

### 2. DK Group Mice Exhibit Less Weight Gain but Similar Plasma Lipid Levels Compared to AK Group Mice

After 12 weeks of being fed the HFD, the weight gain of the DK group was much lower than that of the AK group mice (*Table 1*). In order to eliminate the influence of amouts of food on weight gain, the daily food intake was also noted and showed little alteration between groups, indicating that changes in weight gain could not be attributed to greater food ingestion (*Table 1*).

**Table 1:** Metabolic parameters of different groups of mice fed with a HFD.

Parameters	WT	AK	IK	DK
Body weight at week 0 (g)	21.26±0.94	21.11±0.81	21.44±1.04	21.38±1.02
Body weight at week 12 (g)	32.41±1.09	37.44±2.12* #	31.55±0.53△	32.66±0.87△
TG (mmol/L)	1.58±0.68	3.31±0.69* #	1.17±0.48△	3.49±0.53* #
TC(mmol/L)	5.88±0.71	20.28±2.54* #	5.01±0.55△	22.71±2.12* #
Gram food intake per body weight(g/g)	0.105±0.053	0.121±0.044	0.108±0.054	0.115±0.052
LDL(mmol/L)	0.85±0.22	6.23±1.47* #	0.58±0.37△	6.44±1.55* #
HDL(mmol/L)	1.73±0.87	0.68±0.14* #	2.16±0.68△	0.57±0.19* #

Body weight was measured at the beginning and after 12 weeks of exposure to a HFD. Serum TC, LDL, HDL and TG levels were measured after 12 weeks of exposure to a HFD. Gram food intake per body weight was measured every morning through the weighing of the HFD, and mice of each group were measured at three different time points from week 0, 6 to 12 to obtain the average food intake. Values are means ± SD; n = 8 per group.

* P<0.05 vs. WT;

# P<0.05 vs. IK;

△P<0.05 vs. AK.

While no significant serum lipid alterations were observed between the WT and IK groups, significant increases in the serum TC and TG levels of the AK and DK groups were found. However, no notable increase in the level of either serum TC or TG was observed between the AK and DK groups (*Table 1*). At the same time, the low density lipoprotein (LDL) measurements also followed the same trends as that of TC and TG, while the high density lipoprotein (HDL) levels were totally reversed (Table 1).

### 3. IKKε Knockout Mice Show Reduced Morphological Changes and Lipid Accumulation in the Aorta After HFD Feeding

**Figure 2:**
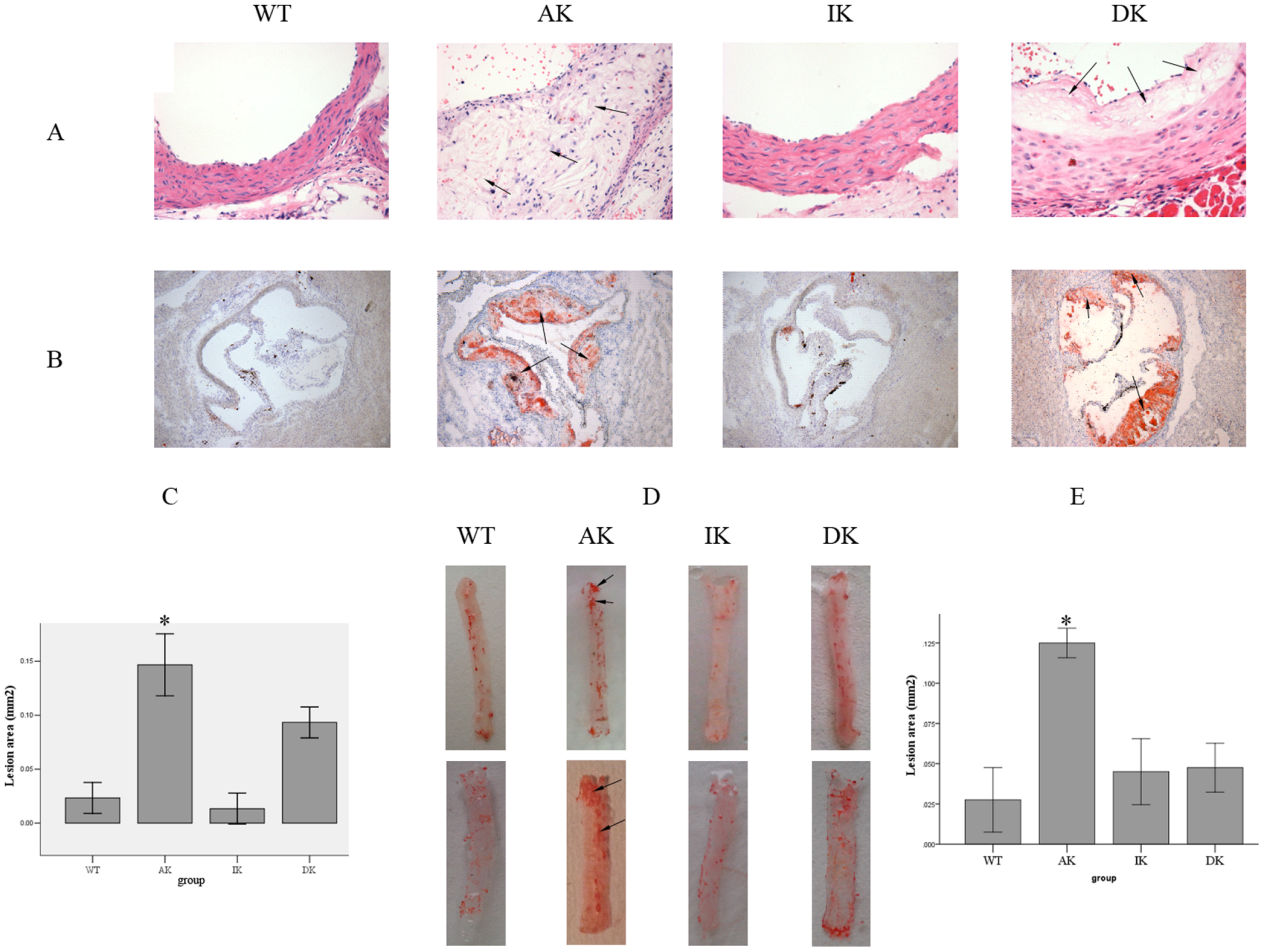
Morphological changes and lipid accumulation of aorta after a HFD feeding. (A) H&E staining of the aortic vessel wall (8 per group) of the 4 groups of mice. Atherosclerotic lesions, lipid vacuolation and cholesterol crystallization are marked with arrows (400×). (B) Oil Red O stained lipid droplets in frozen aortic vessel wall sections (8 per group) from 4 different groups of mice. Lipid accumulations are marked with arrows (100×). (C) Quantitation of the mean Oil Red O stained plaque areas in the frozen aortic root vessel wall sections (8 per group). Values are means ± SD; n = 8 per group; *P<0.05. (D) Oil Red O staining of the total length of thoracic aorta (8 per group) from 4 different groups of mice after HFD exposure for 12 weeks. Lipid accumulations are marked with arrows. (D) Quantitation of the mean Oil Red O stained plaque area in total length of thoracic aorta area. Values are means ± SD; n = 8 per group; *P<0.05.

Atherosclerotic lesion development was assessed by H&E staining of the aortic root in serial paraffin embedded sections (*Figure 2A*). In the DK group, the intimal integrity was maintained, and the media and adventitia still appeared to be arranged in an orderly fashion despite the atherosclerotic lesions in the intima. Compared to that of the DK group, the intimal integrity was destroyed and the intima thickness was increased greatly in the AK group, which most notably exhibited the formation of thicker and larger atherosclerotic lesions, lipid vacuolation and cholesterol crystallization in the vessel wall. Virtually none of the different types of cells was observed in an orderly arrangement in either the media or adventitia.

Oil O Red stained frozen cryosections of the.aortic root area were used to investigate lipid accumulation among different groups of mice. The DK group had less Oil O Red staining in the aortic root area, compared with the AK group of mice, but at the same time showed considerably more staining than the WT and IK groups (Figure 2B). This result was further confirmed by quantitation of the plaque areas (Figure 2C). Oil O Red staining of the total length of the aorta vielded similar results (Figure 2D), which was also validated by quantitation of the plaque areas (Figure 2E). While two representative aortas per group are shown in Figure 2, the remaining eight aortas are provided in the Supplemental data (*Figure S2*).

**Figure 3:**
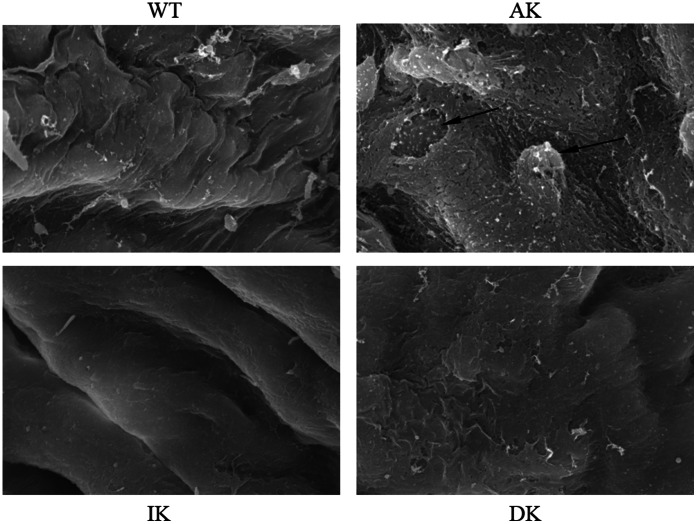
Morphological changes of mouse aortic vessel wall of each group at the subcellular level observed by the scanning electron microscopy. Monocytes adhering and migrating to the endothelial cells and the incomplete endothelium are marked with arrows (3000×). Ten samples were chosen randomly from each group of mice with similar results. Each sample of different mice were repeated three times with similar results.

Morphological changes of the aortic vessel wall were also investigated at the subcellular level by scanning electron microscopy (*Figure 3*). After 12 weeks of exposure to a HFD, the aortic endothelium of the DK group remained intact and without injury, while parts of the aortic endothelium were stripped and the integrity of the intima destroyed in the AK group. Another distinct observation in the AK group was that the monocytes were seen adhering and migrating to the endothelial cells, which is an important event in the initiation of early atherosclerosis as the monocytes further differentiate into macrophages and foam cells in the intima. Meanwhile, the DK group did not exhibit the morphological changes described above.

### 4. IKKε Knockout Mice Inhibit the Expression of NF-κB Pathway Components in the Aorta

**Figure 4:**
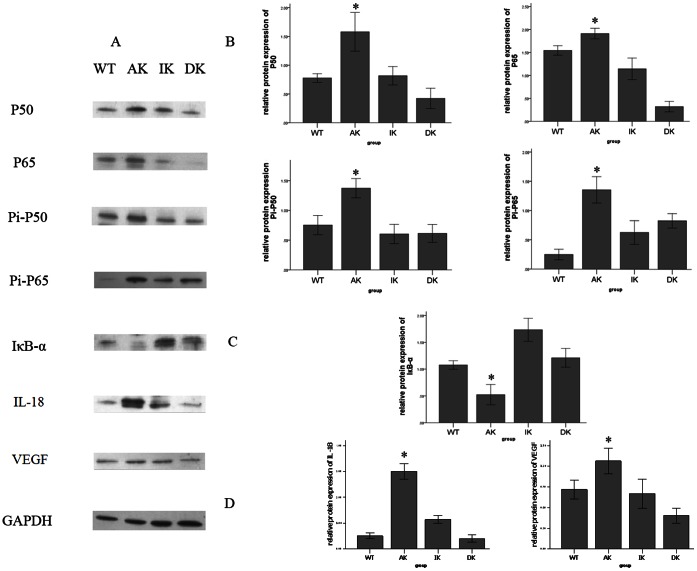
Protein expression of NF-κB cascade components determined by Western blotting. (*A*) Protein levels of IKB-α, P50, P65, phosphorylated p50 (Pi-P50), phosphorylated p65 (Pi-P65), IL-18 and VEGF were measured by Western blotting. (*B*) Expression levels of P50, P65, Pi-P50 and Pi-P65 in the aortic vessel wall, after normalization to GAPDH were all significantly decreased in the DK group compared to the AK group of mice. (*C*) Expression of IKB-α normalized to GAPDH in the aortic vessel wall was greatly increased in the DK group compared with the AK group. (*D*) Expression of both NF-κB downstream factors, IL-18 and VEGF were downregulated in the DK group compared with the AK group. Values are means ± SD; n = 9 per group. Densitometric data are from one representative experiment of three separate experiments. *P<0.05.

By Western blot analysis, expression levels of both P65 and P50, two important elements which normally form the most common heterodimers of NF-κB, were shown to be significantly reduced in the DK group compared to the AK group. Meanwhile, the expression levels of P50 and P65 in the DK group remained at the same low levels as those of the WT and IK groups (*Figure 4A–B*). Phosphorylated P65 and P50, comprising the functionally active form of the transcription factor in the nucleus, and IκB-α, which binds with NF-κB to inhibit its activation, were also detected. The results show that the expression of phophorylated P65 and P50 (*Figure 4B*) followed the same trend as that of total P65 and P50, while the expression pattern of IκB-α (*Figure 4C*) was reversed among the four groups of mice. The protein expression of IκB-α was highly increased in the DK group compared to the AK group, while that of the other two groups of mice stayed at the same level as the AK group. In addition, the expression levels of two NF-κB pathway downstream factors, IL-18 and VEGF, were also downregulated in the DK group compared with the AK group (*Figure 4D*). One representative result per group is shown in Figure 3, while the remaining blots are given in supplemental data (*Figure S3*).

### 5. Expression of NF-κB Pathway Components are Activated by IKKε and Localize in Nuclei of Cells in the Intima Area of Aortic Vessel Wall

**Figure 5:**
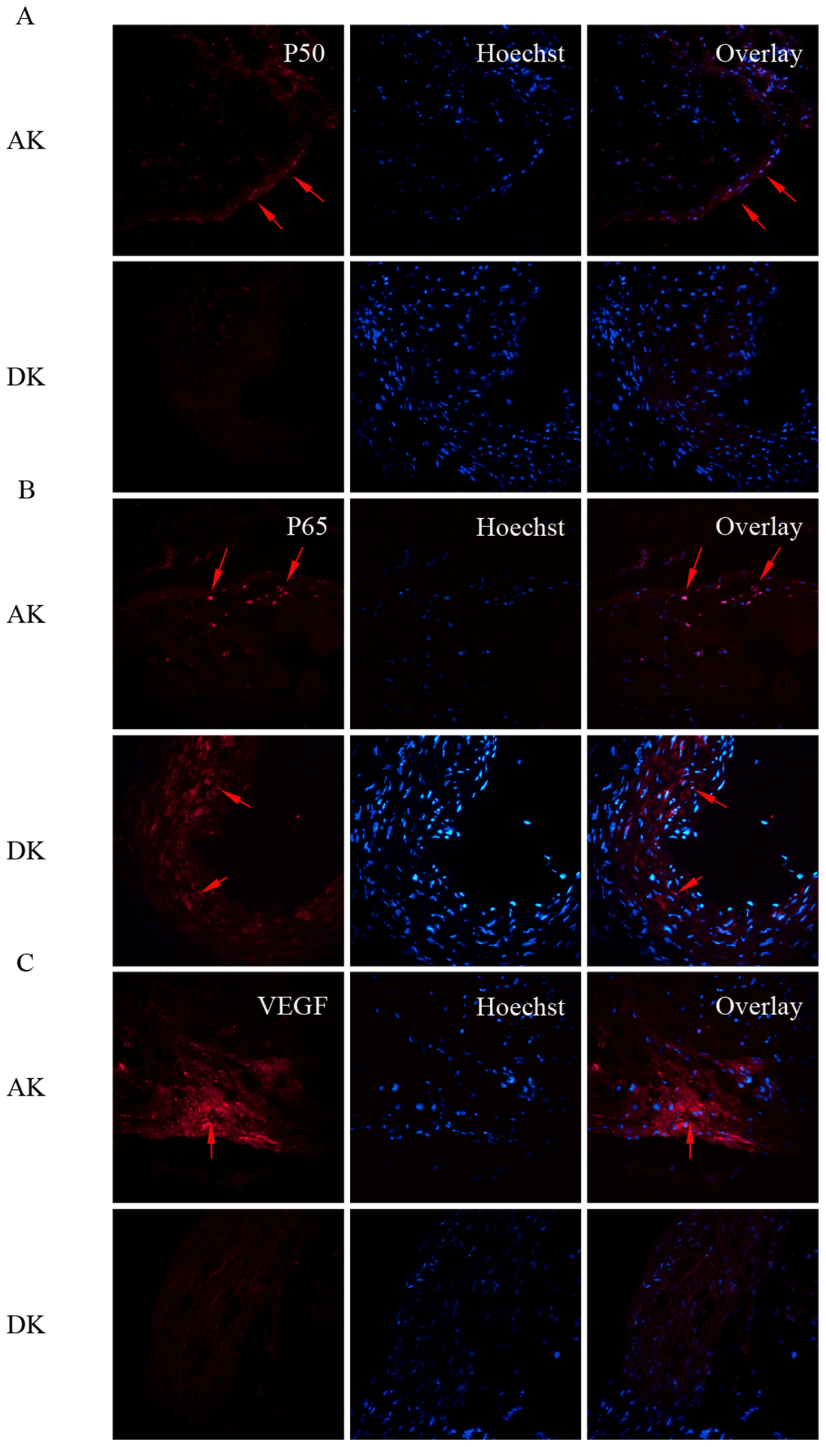
Protein expression of NF-κB cascade components determined by immunofluorescence. Representative images show merged nuclei (Hoechst, blue)+P50-positive staining (A, red), nuclei (Hoechst, blue)+P65-positive staining (B, red) and nuclei (Hoechst, blue) +VEGF-positive staining (C, red). The positively stained areas are marked with arrows (400×); n = 8 per group.

By immunofluorescence analysis of the aortic root area, we next examined the expression and localization of P65, P50 and the NF-κB pathway downstream factor IL-18 in the aortic vessel wall. The expression levels of both P65 and P50 were low in the aorta of the DK group compared with the AK group. Interestingly, the localization of both P65 and P50 was widely spread throughout the cytoplasm of all different cell types of the aortic vessel wall in the DK group. At the same time, the localization of these proteins transferred to the nuclei, especially in the intima area, indicating that most of the activated form of the nuclear factor shifted to the nuclei of the intima to take effect as a downstream target gene regulator in the AK group (Figure 5A,B). In addition, the NF-κB pathway downstream factors IL-18 was also downregulated in the DK group compared with the AK group (Figure 5C).

## Discussion

Recent studies have suggested a key role for the NF-κB pathway in the development of atherosclerosis [2], [3], [8], [9]. The NF-κB family is a transcription factor family consisting of five subunits, including RelA (p65), RelB, cRel, P50 and P52, which can homo- and hetero-dimerize through their conserved prototypical Rel homology domain (RHD). These members bind with the κB sites of target genes to regulate their expression and subsequent biological effects. Among the different types of NF-κB dimers, the P65/P50 heterodimer is the most common form and plays the most important role in the activation of downstream targeted genes [24]–[25]. The two principal signaling cascades, the canonical pathway and the alternative pathway, are each activated by different components of the IKK complex [26]. IKKβ mainly participates in the canonical pathway [27], which plays critical roles in multiple processes affecting health and disease, including immunity, inflammation, cell growth, stress and cell survival [28]. Since the role of IKKβ in atherogenesis is still controversial [15]–[16], we shifted our focus to other members of the IKK complex that can activate NF-κB signaling. Inspired by recent studies indicating that IKKε, which shares 31% amino acid identity with IKKβ in the highly conserved N-terminal kinase domain, activates transcription factors such as NF-κB, interferon regulatory factor-3 (IRF3) and CCAAA/enhancer-binding protein (C/EBPδ) to modulate such processes as inflammation, antiviral immunity and cell survival [21], [29], we discovered a close relationship between IKKε and HFD-induced athersclerosis through the NF-κB pathway.

To confirm that the activation of IKKε promotes the HFD-induced atherosclerosis in mice, immunohistochemical staining of IKKε was performed in mouse aortic vessel walls and showed a dramatically increased expression, especially in the intima area rather than the adventitia or media in the AK group compared to the WT group. Consistent with results of immunohistochemical staining, the Western blot analysis revealed an upregulation of IKKε in the AK group, which implied that HFD-induced pathological and morphological changes may be attributed to the upregulation of IKKε.

We next examined the general metabolic changes to assess the influence of the HFD on body weight and serum lipid profile by employing ApoE knockout mice, which spontaneously develop atherosclerotic lesions with an elevated serum lipid level [30]. Consistent with the former finding that a HFD led to the sustained elevation of body weight and lipid profile, we found a dramatic increase in weight gain and serum lipid level in the AK group compared to the WT group. While the increased weight gain was completely blocked in the DK group, serum lipid levels were not significantly different between the AK and DK groups. These results suggest an inhibitory role for IKKε in HFD-induced obesity. At the same time, IKKε did not affect the serum lipids notably, which excluded the potential role for lipid alteration rather than IKKε knockout in the HFD-induced morphological and molecular changes. In contrast, an independent study found that IKKε knockout had no effect on inflammation in major metabolic tissues and on insulin resistance when the anti-obesity effect of the IKKε knockout was overridden by a more aggressive HFD regimen [31]. Thus, the inflammatory effects of IKKε in metabolic disease are apparently not independent of its obesigenic effects. This view is further strengthened by a very recent work demonstrating the anti-obesigenic effects of IKKε and TBK1 inhibitors [32]. From a review of the relevant studies in the literature, we found contradictory conclusions on the effect of IKKε knockout on body weight regulation, inflammation and insulin resistance. It seems that the regulation of weight gain on a HFD may be one of the causes for reduced NF-kB signaling and atherosclerosis. In our study, the most significant metabolic effect of IKKε knockout was also the prevention of weight gain on a HFD, and this could have been at least one of the causes for reduced NF-kB signaling and atherosclerosis in the DK mice. Thus, a limitation of our study was not being able to avoid the influence of the prevention of weight gain on the reduced NF-kB signaling and atherosclerosis in the DK mice. In spite of that, we could still reach an agreement that IKKε is a bridge between obesity and inflammation [33] and in many inflammatory diseases IKKε takes effect through the activation of NF-kB pathway signaling [22]–[23]. Further investigation is warranted to clarify the direct regulatory mechanism between IKKε and atherosclerosis.

We also examined the morphology of aortas to determine the pathological changes brought about by a HFD and whether they could be reversed by knockout of IKKε. Indeed, severe pathogical changes were induced by the HFD as described above with the application of H&E and Oil Red O staining. By scanning electron microscopy, we obtained a distinct view of the ultrastructural alterations of the aortic vessel wall in the DK group compared with the AK group. Collectively, these data illustrate a comprehensive picture of the HFD-induced pathological changes from the whole aortic vessel wall tissue to the subcellular level, while IKKε knockout mice were protected from all these alterations. Thus, IKKε is a potential regulatory factor in HFD-induced atherosclerotic lesions.

We also examined changes in major components of the NF-κB cascade in the aortic vessel wall among different groups of mice to determine whether the IKKε knockout mice were protected against the damage due to the HFD through the inactivation of NF-κB [34]–[35]. The expression levels of total and phosphorylated P65/P50 in the mouse aortic vessel wall were all downregulated in the DK group compared with the AK group. Another surprising finding is that, unlike the other components of the NF-κB cascade, the decrease in expression of IκB-α in the AK group was reversed in the DK group. This result could be explained by the HFD inducing the NF-κB cascade through the phosphorylation and ubiquitination of IκB-α to release NF-κB for its translocation to the nucleus. Since there was more degradation of IκB-α, the expression of the IκB-α protein would also certainly decrease, as confirmed by previous studies [36]. Furthermore, we also gained a better understanding of the downstream inflammatory cytokines regulated by activation of the NF-κB cascade in the initiation and progression of atherosclerosis. IL-18, an NF-κB-activated proinflammatory cytokine, has been demonstrated to promote the development of atherosclerosis [37]. VEGF is also known as a downstream factor of the NF-κB cascade which plays vital roles in the neovasculization of atherosclerotic lesions [38]. Our assessment of the protein levels of these two downstream proinflammatory factors provided the same results as with the upstream NF-κB components.

The expression and localization of the NF-κB cascade components were also investigated by immunofluorescence in this study, which revealed that P65 and P50 were both upregulated in the mouse aortic vessel wall in the AK group. A striking discovery that deserves noting is that we could distinctly view the translocation of resting NF-κB from the cytoplasm to the nuclei in the intima area, consistent with the manner of NF-κB activation. Furthermore, we also detected VEGF to gain a better understanding of the downstream inflammatory cytokines regulated by activation of the NF-κB cascade in the initiation and progression of atherosclerosis. The expression of VEGF followed the same trend as that of P65 and P50, which provided us with a complete overview of the pathway, starting from the upstream NF-κB to the downstream inflammatory cytokines, and showed that activation of the NF-κB cascades was inhibited in IKKε knockout mice fed with a HFD.

In conclusion, our study suggests that a HFD induces the development of atherosclerosis in the ApoE knockout mouse aortic vessel wall, especially in the intima area, through the IKKε-mediated NF-κB pathway. In the absence of IKKε, the mouse aortic vessel wall is dramatically prevented from sustaining the HFD-induced inflammatory injury and atherosclerosis. This study also provides us with insight into atherosclerosis at the molecular biological level, which may aid in its prevention and treatment.

## Supporting Information

Figure S1 **IKKε detected by Western blotting in the aortic vessel wall of 4 groups of mice.** Expression of IKKε in the AK and WT groups is described in the manuscript. Neither group of knockout mice (IK and DK) showed any expression of IKKε.
(TIF)

Figure S2 **Oil Red O staining of 8 aortas (not shown in main text) of the total length of thoracic aorta from 4 different groups of mice after HFD exposure for 12 weeks.** Lipid accumulations are marked with arrows.
(TIF)

Figure S3 **Blots of protein levels of IKB-a, P50, P65, phosphorylated p50 (Pi-P50), phosphorylated p65 (Pi-P65), IL-18 and VEGF for samples not shown in main text.**
(TIF)
