# Supplementary figures and images for: IKKε Knockout Prevents High Fat Diet Induced Arterial Atherosclerosis and NF-κB Signaling in Mice
Source: PLoS One. 2013 May 31;8(5):e64930. doi: 10.1371/journal.pone.0064930 (PMC3669140; doi:10.1371/journal.pone.0064930)

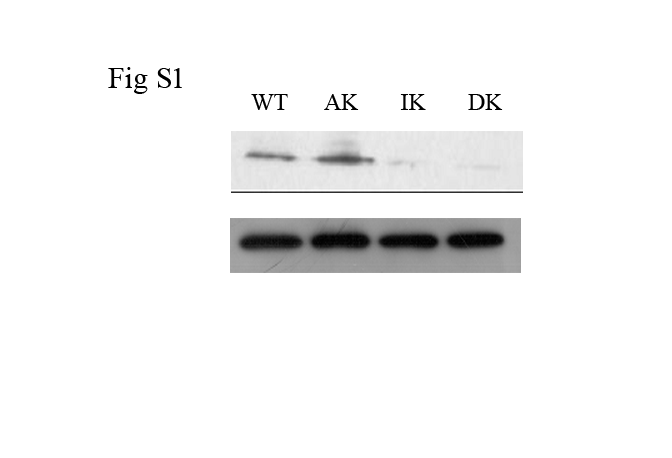

Supplement: Figure S1 — IKKε detected by Western blotting in the aortic vessel wall of 4 groups of mice. Expression of IKKε in the AK and WT groups is described in the manuscript. Neither group of knockout mice (IK and DK) showed any expression of IKKε. (TIF) [file pone.0064930.s001.tif]

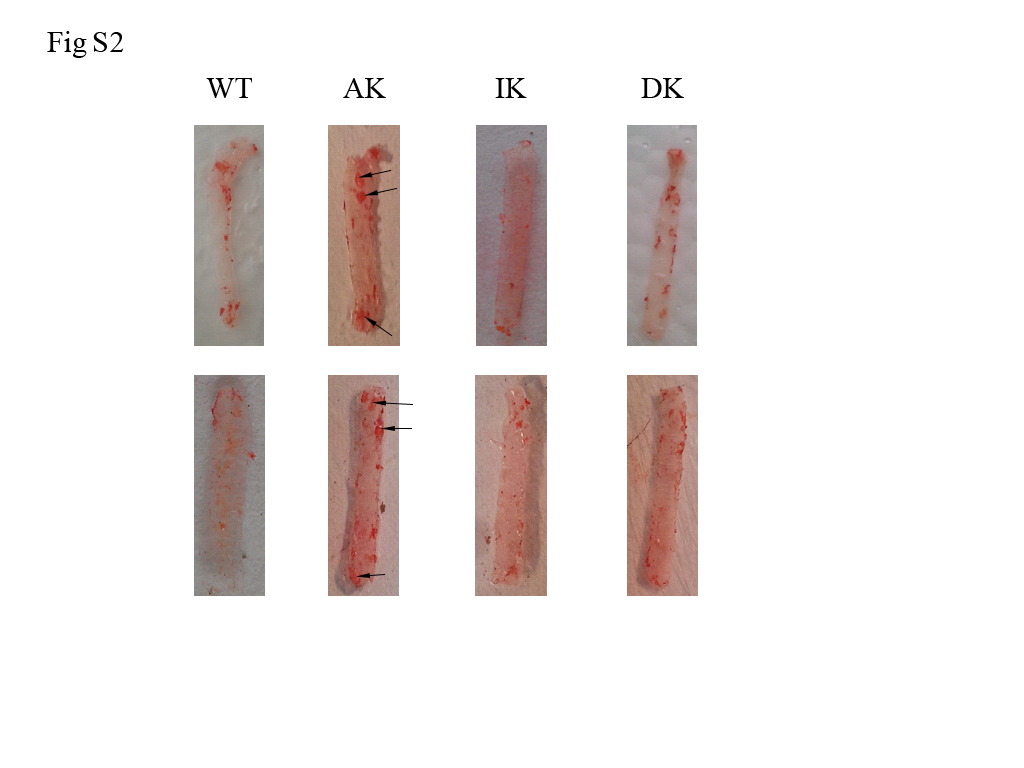

Supplement: Figure S2 — Oil Red O staining of 8 aortas (not shown in main text) of the total length of thoracic aorta from 4 different groups of mice after HFD exposure for 12 weeks. Lipid accumulations are marked with arrows. (TIF) [file pone.0064930.s002.tif]

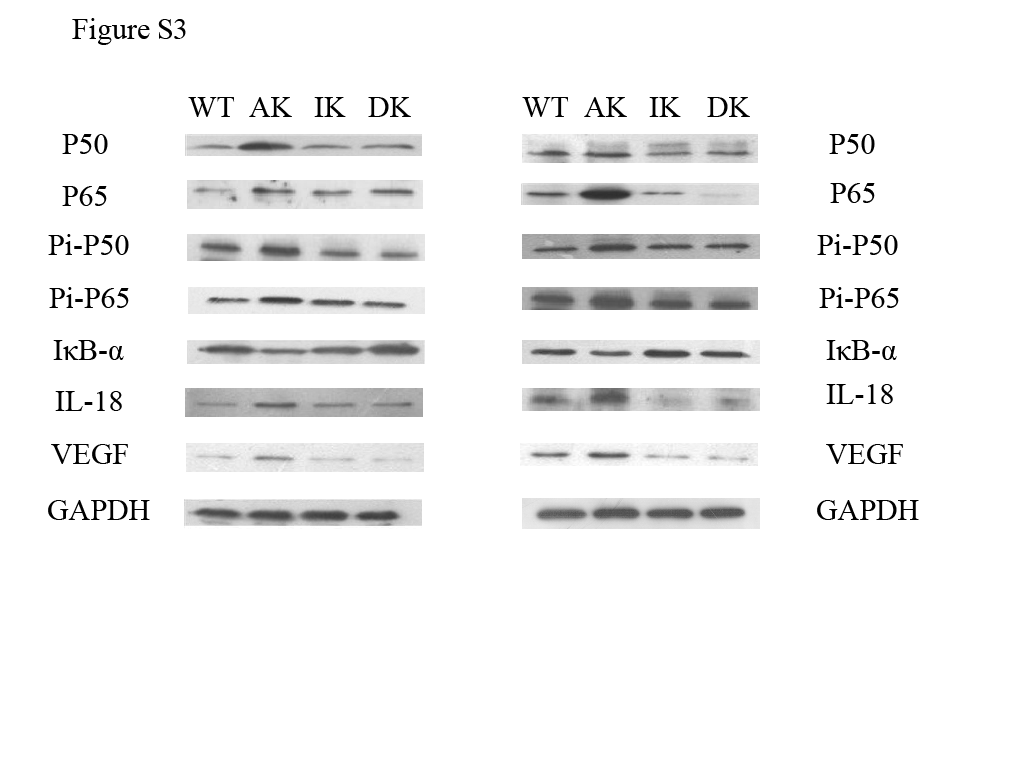

Supplement: Figure S3 — Blots of protein levels of IKB-a, P50, P65, phosphorylated p50 (Pi-P50), phosphorylated p65 (Pi-P65), IL-18 and VEGF for samples not shown in main text. (TIF) [file pone.0064930.s003.tif]
